# Accuracy and Utility of Preoperative Ultrasound-Guided Axillary Lymph Node Biopsy for Invasive Breast Cancer: A Systematic Review and Meta-Analysis

**DOI:** 10.1155/2022/3307627

**Published:** 2022-09-27

**Authors:** Yihong Huang, Shuo Zheng, Yu Lin

**Affiliations:** ^1^Department of Ultrasound, Fuzhou Second Hospital, Fuzhou 350007, China; ^2^Department of Hepatobiliary Surgery, Fuzhou Second Hospital, Fuzhou 350007, China

## Abstract

**Background:**

With the acceleration of the pace of life and work, the incidence rate of invasive breast cancer is getting higher and higher, and early diagnosis is very important. This study screened and analyzed the published literature on ultrasound-guided biopsy of invasive breast cancer and obtained the accuracy and practicality of preoperative biopsy.

**Method:**

The four databases were screened for the literature. There was no requirement for the start date of retrieval, and the deadline was July 2, 2022. Two researchers screened the literature, respectively, and included the literature on preoperative ultrasound-guided biopsy and intraoperative and postoperative pathological diagnosis of invasive breast cancer. The diagnostic data included in the literature were extracted and meta-analyzed with RevMan 5.4 software, and the bias risk map, forest map, and summary receiver operating characteristic curves (SROC) were drawn.

**Results:**

The included 19 studies involved about 18668 patients with invasive breast cancer. The degree of bias of the included literature is low. The distribution range of true positive, false positive, true negative, and false negative in the forest map is large, which may be related to the large difference in the number of patients in each study. Most studies in the SROC curve are at the upper left, indicating that the accuracy of ultrasound-guided axillary biopsy is very high.

**Conclusion:**

For invasive breast cancer, preoperative ultrasound-guided biopsy can accurately predict staging and grading of breast cancer, which has important reference value for surgery and follow-up treatment.

## 1. Introduction

Invasive breast cancer is a kind of malignant tumor [1], and no definite therapeutic factors have been found [2]. With the acceleration of the pace of life and work and the increase of pressure, the incidence rate of invasive breast cancer in women has increased year by year [3]. The main symptoms are painless breast tumors [4]. Early invasive breast cancer can be cured by timely treatment after discovery [5]. After the discovery of advanced invasive breast cancer, timely surgical resection, adjuvant radiotherapy and chemotherapy, and targeted drug therapy can effectively control the progress of the disease [6], with a survival rate of 70% or more. The prognosis is related to the stage of cancer [7]. Breast cancer is divided into invasive breast cancer and noninvasive breast cancer [8]. Invasive breast cancer can invade surrounding tissues and has the ability of distant metastasis [9]. Breast cancer has a high degree of malignancy [10]. According to the World Health Organization (WHO) histological classification of breast tumors [11], invasive breast cancer is divided into special breast cancer and nonspecial breast cancer [12]. Special breast cancer is divided into simple tubular carcinoma, invasive cribriform carcinoma, medullary carcinoma, and mucinous carcinoma [13, 14]. Nonspecific breast cancer is divided into invasive ductal carcinoma and invasive lobular carcinoma [15], of which invasive ductal carcinoma is the most common breast cancer [16, 17], accounting for about 70% to 80% of breast cancer.

At present, no clear therapeutic factors for breast cancer have been found. It is generally believed that pathogenic factors are family history and genetics [18], endogenous estrogen, exogenous estrogen, radiation exposure, benign breast disease and breast cancer in situ, alcohol intake, caffeine intake through coffee and tea [3], weight gain or overweight, sedentary, diet dominated by meat and sweets, and insufficient vitamin intake [19, 20]. Early detection and treatment are very important [21]. The grading and staging of breast cancer has a significant impact on prognosis [22, 23]. The WHO takes the Nottingham grading system as the standard histological grading system for invasive breast cancer [24, 25]. The evaluation indicators are the proportion of glandular duct formation, nuclear pleomorphism, and mitotic image count [26]. The widely used biopsy method for the diagnosis of breast cancer is ultrasound-guided axillary lymph node biopsy, observing the removed tissue under the microscope to make an accurate diagnosis [27]. In case of breast cancer, surgical resection treatment should be carried out as soon as possible [18]. This study searched all the literature of PubMed, Embase, Cochrane, and Web of Science, screened out the literature related to preoperative ultrasound-guided axillary lymph node biopsy in patients with invasive breast cancer, and at the same time, identified the results of intraoperative and postoperative pathological diagnosis, meta-analyzed the accuracy and practicality of this biopsy, and drew a meaningful conclusion for clinical treatment.

## 2. Methods

### 2.1. Literature Search

We searched the database PubMed, Embase, Cochrane Library, and Web of Science with Mesh terms and keywords [28]. The Mesh terms were “invasive breast cancer” and “biopsy,” and “invasive breast cancer” includes all the types of invasive breast cancer mentioned above. In order to search the literature as much as possible, we did not take ultrasound-guided biopsy as the subject word. First, we searched all the biopsy literature and then screened and removed the literature that did not use ultrasound-guided biopsy. In PubMed, the Cochrane Library, and the Web of Science database, the search term is ((Invasive breast cancer) OR (Nonspecific invasive breast cancer) OR (Invasive ductal carcinoma) OR (Invasive lobular carcinoma) OR (Special types of invasive breast cancer) OR (Simple tubular carcinoma) OR (Invasive cribriform carcinoma) OR (Medullary carcinoma) OR (Mucinous carcinoma)) AND ((Biopsy) OR (Needle Biopsy) OR (Fine biopsy) OR (Endoscopic Ultrasound-Guided Fine Needle Aspiration) OR (Large-Core Needle Biopsy) OR (core biopsy) OR (Needle Biopsies) OR (Needle Biopsy) OR (Aspiration Biopsy) OR (Aspiration Biopsies) OR (Puncture Biopsy) OR (Puncture Biopsies) OR (Fine-Needle Biopsy)). In the Embase database, the search term is (“invasive breast cancer”: ti OR “nonspecific invasive breast cancer”: ti OR “invasive ductal carcinoma”: ti OR “invasive lobular carcinoma”: ti OR “special types of invasive breast cancer”: ti OR “simple tubular carcinoma”: ti OR “invasive cribriform carcinoma”: ti OR “medullary carcinoma”: ti OR “mucinous carcinoma”: ti) AND (biopsy: ti OR “fine biopsy”: ti OR “endoscopic ultrasound-guided fine needle aspiration”: ti OR “large-core needle biopsy”: ti OR “core biopsy”: ti OR “needle biopsies”: ti OR “needle biopsy”: ti OR “aspiration biopsy”: ti OR “aspiration biopsies”: ti OR “puncture biopsy”: ti OR “puncture biopsies”: ti OR “fine-needle biopsy”: ti). The latest retrieval time is July 2, 2022. There is no time limit for the literature in the database, and the retrieval starts from the earliest establishment time of the database. Two independent researchers searched the database, respectively, screened the literature together, and decided to include the analyzed literature through discussion and consultation. The references included in the literature are further screened to determine whether there are missing documents. There are no language restrictions in searching and screening the literature.

### 2.2. Literature Screening

The retrieved literature was screened according to the inclusion and exclusion criteria. Inclusion criteria were as follows: (1) complete literature can be obtained; (2) the literature mainly describes the diagnosis and treatment of patients with invasive breast cancer; (3) there are preoperative biopsy reports and intraoperative and postoperative pathological diagnosis reports of patients with invasive breast cancer in the literature; (4) preoperative biopsy was axillary lymph node puncture biopsy guided by ultrasound; (5) node puncture biopsy includes ultrasound-guided fine needle aspiration (US-FNA), ultrasound-guided core needle biopsy (US–CNB), and other puncture biopsies; (6) the literature has no language restrictions and no publication time restrictions.

Exclusion criteria were as follows: (1) duplicate literature; (2) review literature; (3) meta-analysis literature; (4) literature on metastasis of primary cancers such as pancreatic cancer, thyroid cancer, liver cancer, gastric cancer, or cervical cancer on the breast and armpit; (5) literature unrelated to preoperative biopsy, such as cryosurgery, chemoradiotherapy, and biopsy technology; (6) unclear description or results and incomplete patient data records.

### 2.3. Data Collection

Two researchers screened the literature and independently extracted the data from the selected literature using structured data collection tables. We extracted the basic data of patients with invasive breast cancer included in the literature and the basic situation of the literature. The extracted variables include true positive, false positive, true negative, and false negative; that is, the results of intraoperative and postoperative pathological diagnosis are compared with the results of preoperative ultrasound-guided axillary lymph node biopsy, and the former shall prevail to obtain the true and false diagnosis results of the latter. The progress information of postoperative patients was extracted, the information of postoperative complications was extracted, and relevant information was extracted from patients with long-term follow-up records.

### 2.4. Statistical Analysis

The Review Manager 5.4 (RevMan 5.4) software of the Cochrane Collaboration Network was used to evaluate the bias risk of the included literature. The specific evaluation contents included the generation of random sequences, allocation concealment, blinding of subjects, blinding of result evaluators, data integrity, and selective reporting. If the opinions assessed by the two researchers were not the same, a third researcher was required to participate in the assessment. RevMan 5.4 software was used to compare the preoperative biopsy results with the intraoperative and postoperative pathological results of all patients with invasive breast cancer included in the literature, and the forest map was made and analyzed. The specificity was studied using 95% confidence interval and calculated using the Mantel– Haenszel random-effect model. Funnel charts were made for all the included literature to visually and clearly reflect the bias.

## 3. Results

### 3.1. Literature Search and Screening Results and Basic Information of the Included Literature

**Figure 1:**
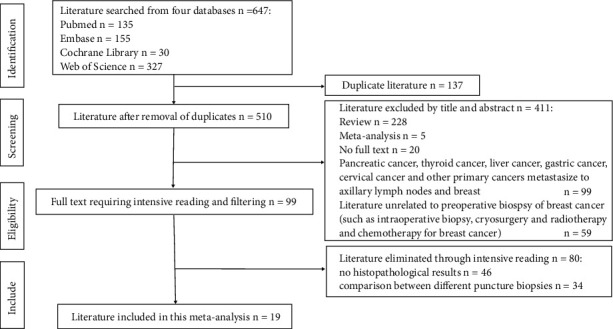
The literature screening flowchart. 647 articles were retrieved from four databases, and 22 articles were included in meta-analysis after screening.

Four databases were searched, and a total of 647 documents were obtained. Among them, there are 135 PubMed, 155 Embase, 30 Cochrane Library, and 327 web of science. Among them, 137 literature reviews were repeated. After reading the title and abstract, combined with the conditions of inclusion and exclusion of documents, 411 documents were eliminated. After intensive reading of the articles, 80 articles were eliminated, and 19 articles were finally included in meta-analysis. The flowchart of literature screening is shown in Figure 1.

The authors, countries, languages, the number of patients with invasive breast cancer, and types of preoperative ultrasound-guided biopsy of 19 literature reviews were extracted and listed. As shown in Table 1, the total number of patients with invasive breast cancer included in the literature was about 18668. All the above work was completed by two researchers.

### 3.2. Bias-Risk Assessment of Included Articles

**Figure 2:**
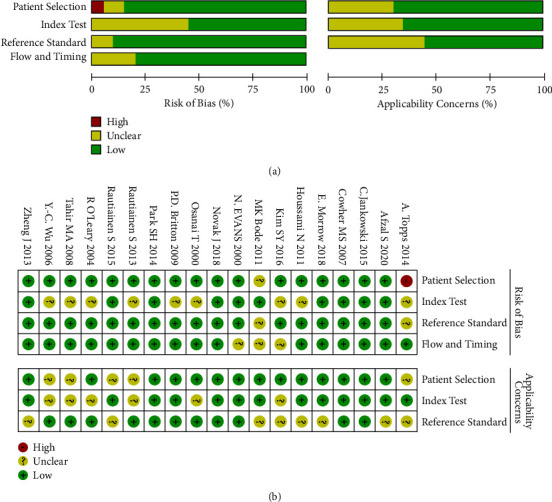
The bias of 20 included literature reviews is drawn with RevMan 5.4. (a) Risk of bias and applicability concerns graph: review authors' judgements about each domain presented as percentages across included studies. (b) Risk of bias and applicability concerns summary: review authors' judgements about each domain for each included study.

RevMan 5.4 software was used to analyze the bias of 20 included literature reviews. The literature reviews were analyzed from four aspects: patient selection, index test, reference standard, and flow and timing (see Figure 2 for details).

### 3.3. Forest Plot

**Figure 3:**
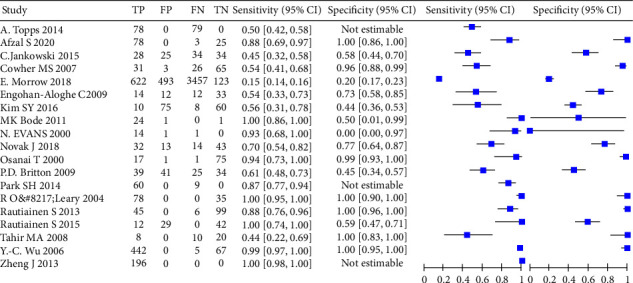
Forest plot. Values are shown with 95 percent confidence interval.

The forest map was drawn with RevMan 5.4 software, and false positive, false negative, true positive, and true negative of 19 studies were counted (see Figure 3 for details).

### 3.4. SROC Curve

**Figure 4:**
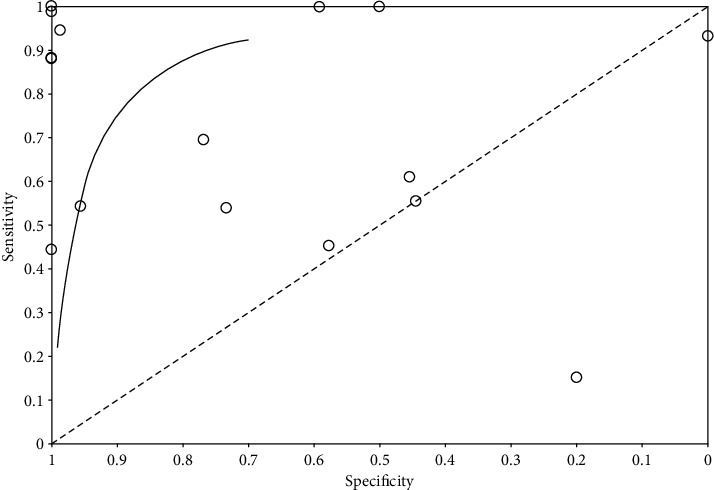
SROC curve.

Using RevMan 5.4 software to draw the SROC curve, it is found that most studies are distributed in the upper left, some of which are close to 1, and only two studies are distributed in the lower left (see Figure 4).

## 4. Discussion

Invasive breast cancer metastasis to axillary lymph nodes can help determine the stage of invasive breast cancer. Through imaging examination of other parts of the body, if no metastasis is found, it is determined to be in the early stage of breast cancer. Lymph node metastasis in breast cancer is usually stage 2 or more. After the tumor focus of breast cancer metastasizes to ipsilateral axillary lymph nodes, it can still be pushed locally, indicating that it has entered stage 2 breast cancer. Stage 3 of breast cancer will present after tumor foci have metastasized to the ipsilateral axillary lymph nodes. Metastasis develops to supraclavicular lymph node metastasis, and the patient also has distant organ metastasis, which indicates that it is stage 4 of breast cancer. Ultrasound-guided axillary lymph node biopsy can make a more accurate judgment on the staging of breast cancer, so as to guide the treatment method and the surgical resection scope. At present, there are many new biopsy technologies, such as nuclear magnetic resonance-guided biopsy, and ultrasound-guided biopsy is the most widely used and longest used biopsy technology. There is an urgent need to summarize and analyze the accuracy and applicability of this technology to provide guidance for clinical biopsy of breast cancer.

In this study, 19 literature reviews were selected to compare the preoperative ultrasound-guided axillary biopsy of invasive breast cancer with the intraoperative and postoperative pathological results, and we found out the number of false positive, false negative, true positive, and true negative and drew the forest map and SROC curve. The results showed that most of the studies had high sensitivity and specificity, most of the studies were on the upper left of the SROC curve, and some of these studies are close to 1, indicating that preoperative ultrasound biopsy has high diagnostic accuracy and can effectively predict the metastasis of breast cancer.

In recent years, there have been many new techniques for preoperative biopsy of breast cancer. The application of imaging in the diagnosis of breast cancer is becoming more and more mature, especially the application of ultrasound technology. Chung et al. [47] compared the role of ultrasound, CT, MRI, and PET/CT in predicting axillary lymph node metastasis in breast cancer. 1472 patients with invasive breast cancer with ultrasonic staging of lymph nodes were examined by the above nonultrasonic examination. By comparing with the status of biopsy lymph nodes, it is concluded that the accuracy of ultrasound diagnosis of the supraclavicular region, suspicious supraclavicular lymph nodes, and the IM region is more than 93%, and the overall accuracy of other imaging examinations is lower than that of ultrasound. Zhang et al. [48] compared three ultrasound techniques to detect and predict the risk of axillary lymph node (AlN) metastasis of breast invasive ductal carcinoma. They found that when conventional ultrasound (C-US), ultrasonic elastography (UE), and percutaneous contrast-enhanced ultrasound (P-CUES) were combined, their sensitivity, specificity, positive predictive value, and negative predictive value were 94%, 89%, 86%, and 95%, respectively, which were higher than the detection and prediction results.

It can be seen that ultrasound technology itself can accurately and clearly diagnose breast cancer, while biopsy guided by gold standard ultrasound and histopathological analysis can make an accurate diagnosis of malignant invasive breast cancer. Ji et al. [49] evaluated metastasis of breast lymph nodes in breast cancer by ultrasound-guided core real needle biopsy (CNB). The results showed that 131 of the 164 internal mammary lymph nodes treated with CNB were confirmed to be metastasis positive by histopathology, where 8 were negative and 25 were in an unknown state, indicating that ultrasound can accurately detect lymph nodes that may be malignant. Real time ultrasound-guided CNB and fine needle biopsy (FNA) are accurate and valuable techniques to determine the condition of breast cancer. Wahab et al. [50] conducted a meta-analysis on pure flat epithelial atypical (FEA) lesions diagnosed in core needle biopsy (CNB). The results showed that when the combined escalation rate of breast cancer was 5%, pure FEA diagnosed by CNB should be surgically removed. If more than 90% of the targeted calcification was removed by CNB, close imaging follow-up was recommended. Shehata et al. [51] conducted a meta-analysis on the risk of upgrading to malignant tumors after the diagnosis of a lobular tumor by core needle biopsy of the breast. Through reading and summarizing a large number of literature reviews, it was concluded that the risk of upgrading to malignant tumors was less than 45%, and the risk was low. It was speculated that imaging examination was likely to be an alternative method of surgery. Song [52] performed a meta-analysis on the accuracy of targeted axillary lymph node biopsy (TLNB) in breast cancer patients with positive initial lymph nodes. Regression analysis showed that the overlap of the results of targeted and sentinel lymph node biopsy may be related to the identification rate (IFR) and the false negative rate (FNR), while the new technology TLNB has good IFR, low FNR, and high NPV. On the other hand, the relationship between breast cancer and other tumors also deserves attention [53].

In this study, the preoperative ultrasound-guided biopsy of invasive breast cancer was meta-analyzed and compared with the final diagnostic results. There are some limitations in this study that need to be supplemented by subsequent research. The literature searched is still small, so we should search other databases such as clinical trials and national libraries of various countries. It is necessary to use the full-text search function to retrieve the literature related to preoperative ultrasound biopsy. There may be some literature related to relevant content, but it is not the subject content of the literature. This kind of literature should also be carefully read and screened, and there may be some gains. Ultrasound-guided biopsy is the most widely used. In recent years, MRI-guided biopsy and *X*-ray interventional biopsy have appeared, which can compare various imaging biopsies and draw clinically meaningful conclusions. Preoperative biopsies of different types of invasive breast cancer can be studied to find the differences, such as the location of invaded axillary lymph nodes, so as to conduct in-depth research on invasive breast cancer [54, 55].

## 5. Conclusions

For invasive breast cancer, preoperative ultrasound-guided axillary lymph node biopsy can accurately predict the grading and staging of breast cancer, with an accuracy of more than 95%, which can provide a reference for surgery. The histopathological examination of the tumor during and after the operation was highly consistent with the preoperative biopsy, which confirmed that the accuracy of ultrasound-guided biopsy was very high. At present, ultrasound-guided biopsy of breast cancer is the most widely used technology, and other imaging methods cannot compete with it. Preoperative ultrasound-guided biopsy plays a key role in the operation and treatment of breast cancer or even a decisive role.

## Figures and Tables

**Table 1:** Basic information and the patient information in the literature.

	Country	Language	Number of patients	Preoperative ultrasound-guided biopsy
Osanai [29]	Japan	English	31	US-CNB
Britton et al. [30]	U.K.	English	142	CB, SLN, ALND
Engohan-Aloghe et al. [31]	Belgium	English	71	US, ALND, USG-FNA
Evans and Lyons [32]	U.K.	English	1562	US
Jankowski et al. [33]	France	English	121	SLNB, ALND
Novak et al. [34]	Slovenia	English	102	US-FNAB, AUS
Morrow et al. [35]	U.K.	English	5076	AUS, FNAC
Kim et al. [36]	Korea	English	142	US, US-FNA
Cowher et al. [37]	U.S.A	English	152	AUS
Afzal et al. [38]	Pakistan	English	50	SLNB
Bode and Rissanen [39]	Finland	English	25	US, CNB
Wu et al. [40]	Taiwan, China	English	513	US-CNB
O'Leary [41]	—	English	113	CNB
Rautiainen et al. [42]	Finland	English	54	US-CNB
Park et al. [43]	Korea	English	3124	US-14GCNB
Tahir et al. [44]	U.K.	English	197	US-FNAC
Rautiainen et al. [42]	Finland	English	178	US-FNAB, CNB
Topps et al. [45]	U.K.	English	<417	AUS-FNA, AUS-SNB, AUS-ALND
Zheng et al. [46]	Canada	English	300	US-CB

*Note.* US-CNB, ultrasound-guided automated percutaneous core needle biopsy. CB, core biopsy. SLN, sentinel lymph node. ALND, axillary lymph node dissection. USG-FNA, ultrasound-guided fine needle aspiration. US, ultrasonography. UNB, core needle biopsy. SLNB, sentinel lymph node biopsy. US-FNAB, ultrasound-guided fine needle aspiration biopsy. AUS, axillary ultrasonography. FNAC, fine needle cytology. US-FNA, ultrasound-guided fine needle aspiration. CNB, core needle biopsy. US-CNB, ultrasound-guided core needle biopsy. US-UNB, ultrasound guided-axillary lymph node core biopsy. US-14GCNB, ultrasound guided-14-gauge core needle biopsy. US-FNAC, ultrasound-guided fine needle aspiration biopsy. US-CNB, ultrasound-guided axillary lymph node core biopsy. AUS-FNA, axillary ultrasound-fine needle aspiration. AUS-SNB, axillary ultrasound-sentinel needle biopsy. AUS-ALND, axillary ultrasound-axillary lymph node dissection. US-CB, ultrasound-guided core biopsy.

## Data Availability

The data used in this study are available from the corresponding author upon request.
